# Annual Address, Delivered before the Philadelphia Medical Society, March 6th, 1860

**Published:** 1860-12

**Authors:** 


Annual Address, delivered before the Philadelphla Medical Society, March 26th, 1860. By R. LaRoche, M D. Published by order of the Society.

This is a neatly printed Pamphlet of 61 pages, and contains a very elaborate and interesting examination of the 5th Satire in the 1st Book of Horace, "in its application to questions of a professional nature." Though a singular theme for an anniversary address, the manner in which it is presented will add to the previously high literary reputation of the author.

